# Miliary tuberculosis with no pulmonary involvement in myelodysplastic syndromes: a curable, yet rarely diagnosed, disease: case report and review of the literature

**DOI:** 10.1186/1476-0711-7-8

**Published:** 2008-03-13

**Authors:** Ioannis K Neonakis, Michael G Alexandrakis, Zoe Gitti, George Tsirakis, Elias Krambovitis, Demetrios A Spandidos

**Affiliations:** 1 Mycobacteriology Laboratory, Department of Clinical Bacteriology, Parasitology, Zoonoses and Geographical Medicine, University Hospital of Heraklion, Voutes, 71201 Heraklion, Greece; 2 Department of Hematology, University Hospital of Heraklion, Voutes, 71201 Heraklion, Greece; 3 Microbiology and Parasitology Laboratory, Department of Veterinary Medicine, School of Health Sciences, University of Thessaly, 224 Trikalon Str., 43100 Karditsa, Greece

## Abstract

**Background:**

Although tuberculosis is not uncommon among patients with myelodysplastic syndrome (MDS), only a few reports of such patients suffering from miliary tuberculosis (MT) exist. MT often presents as a fever of unknown origin and it is a curable disease, yet fatal if left untreated.

**Case presentation:**

We report a case of MT with no clinical or laboratory indications of pulmonary involvement in a patient with MDS, and review the relevant literature. *Mycobacterium tuberculosis *was isolated from the liquid culture of a bone marrow aspirate.

**Conclusion:**

Even if the initial diagnostic investigation for a fever of obscure etiology is negative, MT should not be excluded from the differential diagnosis list. Since it is a curable disease, persistent and vigorous diagnostic efforts are warranted. In suspected cases, mycobacterial blood cultures should be collected as soon as possible after hospital admission and early bone marrow aspirate with mycobacterial cultures is advocated.

## Introduction

The increase in numbers and survival of immunocompromised individuals has contributed to the re-emergence of tuberculosis (TB) as a major public health problem. TB is common among patients with myelodysplastic syndrome (MDS) but only a few reports of such patients suffering from miliary tuberculosis (MT) exist. MT often presents as a fever of unknown origin (FUO). It is a curable disease, yet fatal if left untreated and therefore prompt diagnosis is mandatory.

## Case presentation

A 64-year-old male with refractory anemia with multilineage dysplasia and ringed sideroblasts, was admitted with low grade fever, fatigue and insomnia of three months duration. He was initially diagnosed with MDS five years ago, having a single cytogenetic abnormality of trisomy 19. He was treated with recombinant human erythropoietin and pyridoxine. One year prior to admission he underwent a splenectomy for hypersplenism. Afterwards his hematological parameters improved and he was only occasionally transfused with packed red cells. On admission physical examination revealed low-grade fever (37.5°C) and pallor of the skin. He had normochromic normocytic anemia (hemoglobin 9.8 g/dL), thrombocytopenia (79,000/μL) and a normal white blood count (9,800/μL) with dysplastic neutrophils and mild lymphopenia (1,400/μL). Biochemistry was normal, ESR was 44 mm/h and CRP was 0.75 mg/dl. Chest radiography, urine and blood cultures for common bacteria were negative. A bone marrow aspirate was performed, which revealed a hypercellular bone marrow with multilineage dysplasia, 24% ringed sideroblasts, 2% blasts and no additional cytogenetic abnormalities. Bone marrow trephine biopsy showed areas of hypercellularity without the presence of epithelioid cell granulomas or areas of necrosis. The aspirate was further cultured for mycobacteria. It was inoculated into MB/Bact Blood Culture Bottles for BacT/Alert 3D (bioMerieux, Durham, NC, USA) and Lowenstein-Jensen (LJ) slants (bioMerieux, Marcy l' Etoile, France) and incubated at 37°C. The acid-fast staining of the specimen was positive. The liquid culture turned positive eight days later. The isolate was identified as a member of the *M. tuberculosis *complex with gene probes [AccuProbe (GenProbe, San-Diego, USA)] and further differentiated as *M. tuberculosis *with biochemical analysis. Mycobacterial sputum cultures were negative. The chest computed tomography (CT) scan was normal. Drug susceptibilities were determined with the proportions method. The isolate was susceptible to all anti-tuberculosis drugs tested. The patient received a 4-drug regimen (isoniazid, rifampicin, ethambutol and pyrazinamide) for six months, followed by isoniazid and rifampicin for another three months. He defervesced shortly after the onset of treatment.

## Discussion

The term "Miliary Tuberculosis" refers to all types of progressive disseminated hematogenous TB regardless of the pathological picture [1]. Its incidence is estimated at approximately 1% of all TB cases [2] and it is usually diagnosed when miliary infiltrates are found either on chest roentgenograms and CT scans or when there are autopsy or biopsy findings of miliary organ involvement [1]. However, in some cases such findings may not be evident in the radiological studies and the term "Disseminated Tuberculosis" has been proposed for such, whereas the term "Miliary Tuberculosis" is mostly reserved for cases of disseminated TB with miliary shadows [3].

MDS is a group of clonal myeloid disorders that often progress to acute leukemia [4]. Pancytopenia is a common finding. Cell-mediated immunity is seriously impaired and patients are predisposed to infections, which account for about one third of deaths. In a 10-year review from a comprehensive cancer center, the MDS cases with TB represented 10.5% of the total number of patients diagnosed with TB and hematological malignancies [5]. MT was reported in 3% of the cases of TB with malignancy. However, there was no report on whether MDS patients were included in this percentage. In a recent report regarding patients with hematological malignancies and TB, 7.2% of them suffered from MDS, but none of the TB cases was reported as miliary [6]. The authors commented that MT was considered as rare in the population studied. In another study, the frequency of TB among MDS patients was found to be 7.7% [7]. Among those cases only one was reported as miliary according to the radiological pattern, but with no extrapulmonary involvement. Despite this relatively high frequency of TB among MDS patients, only two cases of extrapulmonary tuberculous infections in this population have been reported. The first was a localized tuberculous lymphadenopathy [4], whereas the second was a case of MT in a patient later diagnosed with MDS [8].

MT is a serious illness demanding timely response. Fever is a constant symptom and may take the form of FUO [1]. Other symptoms include malaise, anorexia, weight loss, night sweats, cough, headaches or abdominal pain. In one third of patients, hepatomegaly or splenomegaly can be found [1]. Hematological abnormalities such as chronic disease anemia, leucopenia with lymphopenia, thrombocytopenia or, more rarely, pancytopenia, are frequently described [1,8,9]. It has been suggested that if MT coexists with pancytopenia, the latter may reflect an underlying hematological disease (often leukemia or a preleukemic disorder) [9]. Chest X-ray or CT images may or may not reveal miliary infiltrates, as such lesions need weeks to develop or may not develop at all [1]. In most cases granulomas can be histologically demonstrated in the tissues (lung, liver, lymph node or bone marrow) [10].

Isolation of the mycobacterium provides a definite diagnosis of MT. The use of liquid media cultures has allowed the isolation of *M. tuberculosis *and the determination of its susceptibilities within days. The yield of acid-fast smears is usually low in the investigation of FUO [11,12]. TB is considered to be one of the most common infectious causes of FUO, with high mortality rates [1,9,12], so early diagnosis and treatment is important.

One question raised is whether a physician should perform an early bone marrow aspirate or whether blood cultures are sufficient for the isolation of mycobacteria in probable disseminated cases. In a recent study evaluating the diagnostic yield of blood and bone marrow cultures, it was suggested that disease caused by members of the *M. tuberculosis *complex could be misdiagnosed without a bone marrow culture [13]. The combined use of blood and bone marrow cultures can provide maximum sensitivity for the diagnosis of disseminated mycobacterial infections. If mycobacterial infection is suspected, blood cultures should be collected as soon as possible after hospital admission as they are usually negative when collected later [14].

It is important to emphasize that the forms of TB that most often cause FUO are either the disseminated form without the characteristic miliary pattern or extrapulmonary TB without clear localized features [10]. Physicians usually include TB in their differential diagnosis list when taking care of patients with a prolonged fever of obscure origin. However, in the absence of typical chest X-ray data, and as the initial specimen (usually sputum) acid-fast staining is negative and the clinical manifestations are not specific, TB as an etiology is usually by-passed and the diagnosis (if any) is delayed.

In conclusion, we have reported a rare case of MT with no clinical or laboratory indications of pulmonary involvement in a patient with MDS. Even if the initial diagnostic investigation for fever of obscure origin is negative, MT should not be excluded from the differential diagnosis list. As MT is a treatable disease, persistent and vigorous diagnostic efforts are warranted. In suspected cases, mycobacterial blood cultures should be collected as soon as possible after hospital admission and an early bone marrow aspirate with mycobacterial cultures is advocated.

## Conclusion

• Even if the initial diagnostic investigation for fever of obscure etiology is negative, miliary tuberculosis (MT) should not be excluded from the differential diagnosis list.

• MT is a curable disease, thus persistent and vigorous diagnostic efforts are warranted.

• In suspected cases, mycobacterial blood cultures should be collected as soon as possible after hospital admission and early bone marrow aspirate with mycobacterial cultures should be performed.

## Abbreviations

ESR: Erythrocyte Sedimentation Rate, CRP: C-Reactive Protein.

## Competing interests

The author(s) declare that they have no competing interests.

## Authors' contributions

IN carried out the laboratory procedures and drafted the paper. MA and GT handled the patient clinically. ZG participated in carrying out the laboratory procedures. EK revised the manuscript critically. DS conceived the report, and gave final approval of the submitted version. All authors read and approved the final manuscript.
